# Endogenous IFN-β signaling exerts anti-inflammatory actions in experimentally induced focal cerebral ischemia

**DOI:** 10.1186/s12974-015-0427-0

**Published:** 2015-11-18

**Authors:** Ana R. Inácio, Yawei Liu, Bettina H. Clausen, Martina Svensson, Krzysztof Kucharz, Yiyi Yang, Totte Stankovich, Reza Khorooshi, Kate L. Lambertsen, Shohreh Issazadeh-Navikas, Tomas Deierborg

**Affiliations:** Laboratory for Experimental Brain Research, Department of Clinical Sciences, Lund University, BMC A13, Sölvegatan 17, 22184 Lund, Sweden; Neuroinflammation Unit, Biotech Research and Innovation Centre (BRIC), University of Copenhagen, Ole Maaløes Vej 5, 2200 Copenhagen N, Denmark; Department of Neurobiology Research, Institute of Molecular Medicine, University of Southern Denmark, JB Winsloewsvej 21, st + 25, 2, 5000 Odense C, Denmark; Experimental Neuroinflammation Laboratory, Department of Experimental Medical Sciences, Lund University, BMC B11, Sölvegatan 19, 22184 Lund, Sweden; Present Address: INMED, INSERM U901, Parc Scientifique de Luminy, 163 route de Luminy, BP13, 13273 Marseille cedex 09, France; Present Address: Aix-Marseille Université, UMR S901, 13009 Marseille, France; 7Present Address: Department of Neuroscience and Pharmacology, University of Copenhagen, Copenhagen N, 2200 Denmark

**Keywords:** Cytokines, IFN-α/β receptor, Interferon-β, Inflammatory and immune cells, Knockout mice, Middle cerebral artery occlusion

## Abstract

**Background:**

Interferon (IFN)-β exerts anti-inflammatory effects, coupled to remarkable neurological improvements in multiple sclerosis, a neuroinflammatory condition of the central nervous system. Analogously, it has been hypothesized that IFN-β, by limiting inflammation, decreases neuronal death and promotes functional recovery after stroke. However, the core actions of endogenous IFN-β signaling in stroke are unclear.

**Methods:**

To address this question, we used two clinically relevant models of focal cerebral ischemia, transient and permanent middle cerebral artery occlusion, and two genetically modified mouse lines, lacking either IFN-β or its receptor, the IFN-α/β receptor. Subsets of inflammatory and immune cells isolated from the brain, blood, and spleen were studied using flow cytometry. Sensorimotor deficits were assessed by a modified composite neuroscore, the rotating pole and grip strength tests, and cerebral infarct volumes were given by lack of neuronal nuclei immunoreactivity.

**Results:**

Here, we report alterations in local and systemic inflammation in IFN-β knockout (IFN-βKO) mice over 8 days after induction of focal cerebral ischemia. Notably, IFN-βKO mice showed a higher number of infiltrating leukocytes in the brain 2 days after stroke. Concomitantly, in the blood of IFN-βKO mice, we found a higher percentage of total B cells but a similar percentage of mature and activated B cells, collectively indicating a higher proliferation rate. The additional differential regulation of circulating cytokines and splenic immune cell populations in wild-type and IFN-βKO mice further supports an important immunoregulatory function of IFN-β in stroke. Moreover, we observed a significant weight loss 2–3 days and a reduction in grip strength 2 days after stroke in the IFN-βKO group, while endogenous IFN-β signaling did not affect the infarct volume.

**Conclusions:**

We conclude that endogenous IFN-β signaling attenuates local inflammation, regulates peripheral immune cells, and, thereby, may contribute positively to stroke outcome.

**Electronic supplementary material:**

The online version of this article (doi:10.1186/s12974-015-0427-0) contains supplementary material, which is available to authorized users.

## Background

Stroke elicits a strong inflammatory response that critically contributes not only to tissue demise but also to repair and regeneration and also is thought to underlie the number one secondary complication found in stroke patients: infection [1]. Specifically, the inflammatory response after stroke comprises changes in the expression of inflammatory mediators and immunocompetent cell populations not only locally, in the brain, but also at the blood-brain interface and in peripheral systems, including the spleen. For example, upregulations of interleukin (IL)-1β and CXCL-1 in the brain have been consistently found in experimental models of stroke [2, 3]. Leukocytes infiltrate the brain parenchyma within hours and days after stroke onset, through a compromised blood-brain barrier (BBB) or via active recruitment [2, 4–6]. Pro-inflammatory cascades are rapidly activated following stroke and are paralleled or followed by an immunosuppressant signaling. Within the first days after stroke, while a decrease in pro-inflammatory pathways may occur in the brain [7, 8], immunosuppression is more evident in the periphery. Apoptotic loss of natural killer (NK), B and T lymphocytes in the blood and spleen, a decreased capacity of interferon (IFN)-γ production by blood-derived leukocytes along with atrophy of the spleen and thymus have been described both in stroke patients and/or rodents subjected to stroke and implicated in an increased susceptibility to infection [7–12]. In agreement with the view that limiting post-stroke inflammation will decrease neuronal death or promote neurological recovery, IFN-β has been put forward as a candidate drug for the treatment of stroke (National Institute of Neurological Disorders and Stroke-sponsored phase I clinical trial).

IFN-β is a type I IFN that binds to the IFN-α/β receptor (IFNAR). Systemic administration of recombinant (r)IFN-β is currently used in the treatment of multiple sclerosis (MS), although the exact mechanisms by which IFN-β administration is beneficial in MS are not fully understood [13]. IFN-β may promote a shift from a T helper (T_h_)1 to T_h_2 response by blood-derived cells and reduce the proliferation of T lymphocytes. It also may diminish the entry of inflammatory and immune cells into the central nervous system (CNS) by downregulating adhesion molecules and matrix metalloproteinase expression by leukocytes, as well as by stabilizing endothelial tight junctions at the BBB [13]. In a mouse model of MS, endogenous IFN-β signaling was sufficient to promote a better disease outcome, which was associated with a decrease in CNS inflammation but not with changes in autoreactive T cells [14, 15]. In support, exogenous IFN-β also reduced autoreactive T cell proliferation by inhibiting the antigen-presenting capacity of microglia and astrocytes, the CNS-specific antigen-presenting cells (APCs) [16, 17].

Studies addressing the effects of IFN-β administration in stroke are scarce and divergent. Systemic administration of human rIFN-β before and up to 4 h after induction of stroke in rabbits resulted in neuroprotection [18]. Similarly, rats showed smaller infarct volumes when treated with rat rIFN-β even up to 6 h after stroke [19]. In mice, intracerebroventricular administration of mouse rIFN-β immediately before or after stroke resulted in a decrease in infarct volume at 24 h [20]. However, systemic administration of wild-type (WT) IFN-β or pegylated IFN-β for 3 or 7 days after transient middle cerebral artery occlusion (tMCAo) had no effect on behavioral performance or infarct volume, but aggravated weight loss in rats [21]. In a recent study, IFN-β administration following experimentally induced subarachnoid hemorrhage, in rats, also provided no effect on outcome measures [22].

We investigated the action of endogenous IFN-β signaling on inflammation and on the development of sensorimotor deficits and infarct volume. For that purpose, we used IFN-β and IFNAR knockout mice (IFN-βKO and IFNAR-KO, respectively) and two clinically relevant models of ischemic stroke, tMCAo and permanent middle cerebral artery occlusion (pMCAo). We focused our study on the first 8 days after stroke onset, a time period critical for recovery of neurological function and infarct core formation [23].

## Methods

### Ethical considerations

We conducted animal experiments in accordance with protocols approved by the Malmö/Lund Ethical Committee for Animal Research (M332-09, M243-07) and Danish Animal Health Care Committee (2011/561-1950), as well as to the ARRIVE guidelines. Experiments were carried out in a blinded and randomized fashion.

### Mouse strains and housing conditions

We used 10 to 40-week-old IFN-βKO [24], as well as 8 to 10-week-old IFNAR-KO [14] male mice backcrossed to C57BL/6 for over 20 generations; WT controls (C57BL/6, age- and gender-matched) were purchased from Taconic (Ry, Denmark). Mice were housed in climate-controlled rooms under diurnal conditions, with ad libitum access to water and food.

### Transient middle cerebral artery occlusion

We modeled stroke in mice by tMCAo as described previously [3]. Mice were placed in an incubator at 35 °C during the first 2 h post-surgery and in an incubator at 33 °C overnight. We injected 0.3 mL of 5 % glucose in saline (sterile) subcutaneously, 30 min, 24 h, and thereafter every 12 h up to 4 days after surgery; weight loss ceases typically between days 3 and 4 after tMCAo. We measured body temperature 1 h, 2 h, and daily up to 7 days after surgery. In addition, we assessed body weight before and after surgery on a daily basis.

#### Inclusion criteria

An immediate reduction in regional cerebral blood flow and metabolism (rCBF) upon occlusion of the MCA and reperfusion were set as the primary inclusion criteria (2 of 78 mice were excluded). Moreover, mice that exhibited signs of pain, weakness, and/or distress were euthanatized and included in the mortality rates (one of 76 mice). One IFNAR-KO and one WT control were excluded from the infarct volume analysis due to bad quality of the tissue. Characteristic ipsilateral subcortical and cortical infarcts were verified in all mice [3].

### Permanent middle cerebral artery occlusion

We modeled stroke in mice by pMCAo as reported before [5]. After surgery, mice were allowed to recover from anesthesia in a 28 °C controlled environment and given a subcutaneous injection of 0.05 mg/Kg (body weight) buprenorphine in saline (Temgesic, Schering-Plough, Ballerup, Denmark) three times, every 8 h, starting immediately after surgery.

#### Inclusion criteria

All mice exhibiting a neocortical infarct, assessed using 2,3,5-triphenyltetrazolium chloride (TTC) staining, were included (one mouse of a total of 20 mice was excluded).

### Behavioral tests

#### Composite neuroscore

We evaluated gross sensorimotor deficits 24 h after tMCAo by behavioral tests adapted from [25–27]. Mice were scored with respect to (1) spontaneous rotation (from 0, rotation on the body axis, to 5, no rotation), (2) resistance to lateral force applied to the left, (3) left forelimb flexion on suspension by the tail, (4) left hindlimb flexion when only the hind limbs are lifted from the surface, and (5) forelimb impairment (from 0, paralysis, to 5, normal). For tests 2 to 4, a scoring system of 0 (no resistance or flexion) to 4 (normal) was used; resistance to lateral force applied to the right and right limb flexion also were evaluated. Sham-operated animals scored 32.

#### Rotating pole test

The rotating pole test was performed essentially as described before [3], with a few modifications. Mice were trained to cross the pole at 0, 3, and 10 rotations per minute (rpm), to the left and right, 2 days and 1 day prior to surgery (tMCAo and sham). With respect to the number of foot slips, only the contralateral forelimb was taken into consideration. Scores 2 and 3 were attributed additionally to animals that clearly met the criteria of scores 3 and 4, but fell from the pole, respectively. We video recorded the sessions for a final analysis. All mice were able to cross the pole prior to surgery (scores 5–6). Sham-operated animals scored 5 or 6 at all the modalities, and we did not observe differences between genotypes.

#### Grip strength test

In experiments involving IFN-βKO mice, we used the grip strength test (Bioseb, Vitrolles, France) as reported previously [3]. We present the maximum grip strength value of five trials as percentage of baseline (collected the day before surgery). The grip strength of IFNAR-KO mice and WT counterparts was evaluated as reported previously [28], and we present the maximum grip strength value of five trials (for each paw).

### Carbon black perfusion

The cerebral vasculature was visualized using a protocol described previously [3].

### Isolation of immune and inflammatory cells

Mice were anesthetized using 1.8–2.5 % isoflurane (IsobaVet, Schering-Plough Animal Health, Milton Keynes, UK) in O_2_:N_2_O (30:70). From each mouse, 400 μL of blood was extracted by intracardiac puncture, using heparinized syringes. Blood was gently homogenized and maintained at 4 °C. Thereafter, mice were perfused transcardially with 10 mL of saline (5 mL/min). Brains were quickly dissected, freed from meninges, and kept in Hank’s Balanced Salt Solution (HBSS, Invitrogen, Paisley, UK) supplemented with 0.2 % bovine serum albumin (BSA, Sigma-Aldrich, Deisenhofen, Germany) and 0.01 % ethylenediaminetetraacetic acid (EDTA, Invitrogen) at 4 °C. Spleens were weighed and transferred to 2 % fetal bovine serum (FBS, Invitrogen) in Dulbecco’s modified Eagle medium (DMEM, Invitrogen) at 4 °C. Blood plasma was obtained following standard centrifugation (1300×*g* for 10 min, 4 °C) of blood; plasma was stored at −80 °C.

#### Brain

Ispilateral or contralateral hemispheres of three to four mice of the same genotype (IFN-βKO or WT) were pooled together. Tissue was mechanically dissociated in HBSS supplemented with 0.2 % BSA and 0.01 % EDTA using a Dounce homogenizer and passed through a 40-μm nylon cell strainer (BD Biosciences, Stockholm, Sweden). The cell suspension was centrifuged at 400×*g* for 10 min at room temperature. The resulting pellet was resuspended in 30 % Percoll (GE Healthcare, Sweden) in HBSS and gently overlaid on a 37–70 % Percoll gradient. Following centrifugation at 500×*g* for 20 min at room temperature, cells were collected at the 37–70 % interface and rinsed with 10 % FBS in HBSS. After a last centrifugation at 400×*g* for 10 min at room temperature, the cell pellet was resuspended in 2 % FBS in phosphate-buffered saline (PBS, Invitrogen).

#### Blood and spleen

We added red blood cell (RBC) lysis buffer (eBioscience, San Diego, CA, USA) to 200 μL of blood. We dissociated whole spleens in RBC lysis buffer, using a 40 μm nylon cell strainer (BD Biosciences). We stopped RBC lysis by adding 2 % FBS in PBS. After centrifugation (300×*g* for 5 min at 4 °C), cells were resuspended in 2 % FBS in PBS. Splenocytes were stained with trypan blue and total numbers estimated using a Bürker chamber.

### Flow cytometry

Isolated immune and inflammatory cells were incubated with primary antibodies for 20 min at 4 °C. After rising with 2 % FBS in PBS, cells were incubated with the secondary antibody Streptavidin PErCP (1:200, BD Biosciences) for 20 min at 4 °C (only for detection of CD122). Subsequent to rinsing, cells were exposed to Cytofix for 20 min at 4 °C and rinsed with BD Perm/Wash buffer (both reagents were purchased from BD Biosciences). Finally, we resuspended the cells using 2 % FBS in PBS. Flow cytometry was carried out using a FACSCalibur flow cytometer (BD Biosciences), with analysis being performed using CellQuest (BD Biosciences) for acquisition after exclusion of duplets and FlowJo 8.8.6 (Tree Star, Ashland, OR, USA). For each sample, we analyzed a total of 100,000 events (cells). We present the results as percentage of total cells analyzed, unless otherwise indicated.

We used the following primary antibodies (purchased from BD Biosciences, unless otherwise indicated), each at a dilution of 1:200: B220-FITC, MCHII-PE, CD4-APC, CD25-FITC, CD8-PE, CD122-biotin, CD11b-APC, CD45.2-FITC (BioLegend, USA), and NK1.1-PE.

### Quantification of T_h_1/T_h_2 cytokines protein levels in the blood plasma

We determined the protein concentrations of IFN-γ, IL-1β, IL-10, IL-12, IL-2, IL-4, IL-5, tumor necrosis factor (TNF)-α, and mouse keratinocyte-derived factor (mK or GRO/CXCL-1) in blood plasma by a sandwich immunoassay (Mouse T_h_1/T_h_2 9-Plex Ultra-Sensitive Kit, Meso Scale Discovery, Gaithersburg, MD, USA). We performed the assay according to the manufacturer’s instructions (Ultra-Sensitive Kit). We used 35 μL of undiluted plasma per well (96-well plates). Plates were read using a SECTOR Imager 6000 (Meso Scale Discovery).

### Immunohistochemistry

For a subset of mice, perfusion with saline was followed by perfusion with 45 mL of 4 % formaldehyde in PBS (5 mL/min). Brains were kept in formaldehyde overnight and thereafter in 40 % sucrose in PBS, always at 4 °C. Free-floating, 30-μm-thick coronal brain slices were rinsed three times with PBS and kept in a 5 % blocking solution (5 % normal serum, Jackson ImmunoResearch, Suffolk, UK, and 0.25 % Triton X-100 in PBS) for 1 h, at room temperature. Following blocking, we incubated slices with primary antibody(ies) in 2 % blocking solution overnight at 4 °C. After rinsing, slices were incubated with secondary antibody(ies) in 2 % blocking solution for 2 h at room temperature.

Primary antibodies and respective dilutions used in this study are as follows: rabbit anti-claudin-5 (1:100, Bioworld Technology); rat anti-CD45 (1:500, MCA1031G, AbD Serotec, Europe); rat anti-galectin-3, Gal-3 (biotinylated, 1:500; Acris Antibodies, Herford, Germany); rat anti-Gal-3 (1:1000; kindly provided by Professor Hakon Leffler, Lund University, Sweden); rabbit anti-ionized calcium-binding adapter molecule 1, Iba-1 (1:1000; Wako, Osaka, Japan); and mouse anti-neuronal nuclei, NeuN (1:800; Sigma-Aldrich). The two antibodies targeting Gal-3 yielded equivalent results. Primary antibodies were detected using streptavidin-Alexa488 (Molecular Probes, Invitrogen); anti-rat Cy3; anti-rabbit Cy3; and anti-mouse biotinylated or anti-mouse Cy2; all reagents were diluted 1:400 (antibodies were purchased from Jackson ImmunoResearch). The biotinylated secondary antibody was detected by avidin-HRP (Vector Laboratories, Peterborough, UK). With respect to BBB integrity assays, to detect mouse IgG in the brain parenchyma, we used a specific HRP-conjugated reagent by cell signaling (catalog number 8125), following the instructions provided by the manufacturer. HRP was, in all cases, detected through 3,3′-diaminobenzidine (DAB) precipitates. In some cases, nuclei were counterstained with 4′,6-diamidino-2-phenylindole (DAPI, Sigma-Aldrich).

### Assessment of CD45^+^ cells, in situ

To estimate the number of CD45^+^ cells in the brain, in situ, we performed CD45 immunolabeling, as described above. From each brain, we used four slices with the following stereotaxic coordinates: approximately 0.86, 0.26, −0.34, and −1.34 mm (anterior-posterior, AP) in relation to the bregma. We counted CD45^+^ cells populating one cortical and/or one striatal infarct core region, each corresponding to a field of vision of about 1 mm^2^ per section. Regions were sampled equivalently across mice. Cells were counted under a fluorescent microscope (Nikon Eclipse 80i, Nikon, Solna, Sweden) by an experimenter blinded to the genotype. In addition, CD45^+^ cells were imaged using Nikon A1 Confocal on a Ti-E microscope and data processed using the NIS-Elements software (Nikon).

### Iba-1 and Gal-3 immunoassays

Additionally, we performed Iba-1 and Gal-3 (double) immunolabeling. Here, we used, from each brain, slices corresponding to the following coordinates: approximately 1.10 mm (level 1), 0.50 mm (level 2), and −1.34 mm (level 3) AP in relation to the bregma. Fluorescent dyes were imaged using a Zeiss LSM 510 inverted confocal microscope. For each brain slice, three (within level 1) or four (within levels 2 and 3) Z-stack images with the dimensions of 640 μm × 640 μm × ~40 μm were collected for analysis; one Z-stack image was acquired from the peri-infarct region (negative control for Gal-3) and the remaining from the infarct core.

The density of Iba-1^+^ and Gal3^+^ macrophages/microglia within the infarct core 8 days after tMCAo is relatively high, and cellular processes may overlap. Under these conditions, counting cells is suboptimal, particularly when using epifluorescence microscopy. We therefore quantified the fraction of Iba-1^+^ signal colocalizing with Gal-3 and vice versa by calculating Mander’s coefficients of the acquired Z-stack images (for simplification, referred to as the percentage of Iba-1^+^ cells expressing Gal-3 and vice versa), using ImageJ software (National Institutes of Health, Bethesda, MD, USA), [29]. Signal intensity thresholds were set manually after applying a denoise filter. Our approach minimized false positives by treating each optical slice of each Z-stack independently, instead of analyzing collapsed Z-stack images. Analysis of Gal-3^+^ cell morphology was carried out using Image J. For each brain, we selected 30–40 Gal-3^+^ cells within the infarct core and manually plotted their outline. For each cell, area, perimeter, circularity, Feret’s diameter, and aspect ratio values were calculated. Analysis was carried out on projected Z-stacks, and only non-overlapping cells were included (as determined on non-projected Z-stacks in both XY and Z dimensions).

#### Gal-3 immunoreactivity

We used three additional brain slices per animal (approximately 0.98, 0.38, and −1.06 mm in relation to bregma) for Gal-3 (single) immunolabeling; Gal-3^+^ signal was estimated following a procedure previously described in detail [3].

### Assessment of blood-brain barrier integrity through IgG and claudin-5 immunoassays

When investigating the presence of IgG in the parenchyma of WT and IFN-βKO brains, we used brain slices centered at 0 mm AP in relation to the bregma (equivalent slices were used across animals). The IgG-specific signal within the infarct core and peri-infarct region was, for each section, qualitatively and qualitatively assessed. Adjacent brain slices were used for analysis of claudin-5 immunoreactivity, as described before [30]. Briefly, only claudin-5^+^ vessels showing a relatively strong and continuous (claudin-5) signal were analyzed. The area occupied by and average length of claudin-5^+^ vessels in a selected region of the peri-infarct cortex (1 mm^2^) were quantified, for each section, using the Image J software. All images were acquired using a Nikon Eclipse 80i microscope and the NIS-Elements software.

### Infarct volume

#### IFN-βKO

For each animal, a series of 30-μm-thick coronal brain slices of the entire brain (240 μm between consecutive slices) was used for NeuN immunolabeling; we used equivalent series among animals. Micrographs were acquired with a Nikon Eclipse 80i microscope, using the NIS-Elements software, under standardized conditions. For each slice, the areas corresponding to the contralateral hemisphere (Contra) and to NeuN^+^ tissue within the ipsilateral hemisphere (Ipsi_NeuN_) were encircled using ImageJ. The infarct area (IA) was estimated accounting for edema or tissue shrinkage: IA = Contra − Ipsi_NeuN_. Infarct volume was obtained by volumetric integration.

#### IFNAR-KO

Mice subjected to tMCAO were given an overdose of pentobarbital and fixative-perfused. A series of 30-μm-thick coronal brain slices (180 μm between consecutive slices) was stained with toluidine blue, and infarct volumes were estimated as described in [5]. For mice subjected to pMCAO, brains were sliced into 1-mm-thick coronal slices; slices were stained with TTC as described before [3]. Infarct volumes were estimated as described above for IFN-βKO mice and WT counterparts.

### Statistics

We present the data as mean ± SD, unless otherwise indicated. To compare two groups, we used Student’s *t* test (two-tailed), and comparison of three or more groups was done by two-way analysis of variance (ANOVA), with the following exceptions. For the composite neuroscore and rotating pole scores, we used the Mann-Whitney U test. Mortality rates were assessed using Fisher’s exact test. For IFN-βKO and WT mice undergoing tMCAo or sham surgery, differences in temperature and weight loss post-surgery were analyzed using a mixed model ANOVA, with multiple comparisons being performed for representative time points (24 h, 2, 3, and 7 days). Analysis of the weight before surgery was done using one-way ANOVA. For IFNAR-KO and WT mice subjected to tMCAo, temperature and grip strength asymmetry post-surgery were evaluated using repeated measures ANOVA. All multiple comparisons were performed post hoc using the Student’s *t* test, with Bonferroni correction. Results were considered significant when *p* < 0.05. Unless otherwise specified, in the figures, we present only the results obtained for pair-wise comparisons, with Bonferroni correction.

## Results

### IFN-βKO mice show a higher accumulation of leukocytes in the brain 2 days after tMCAo

**Fig. 1:**
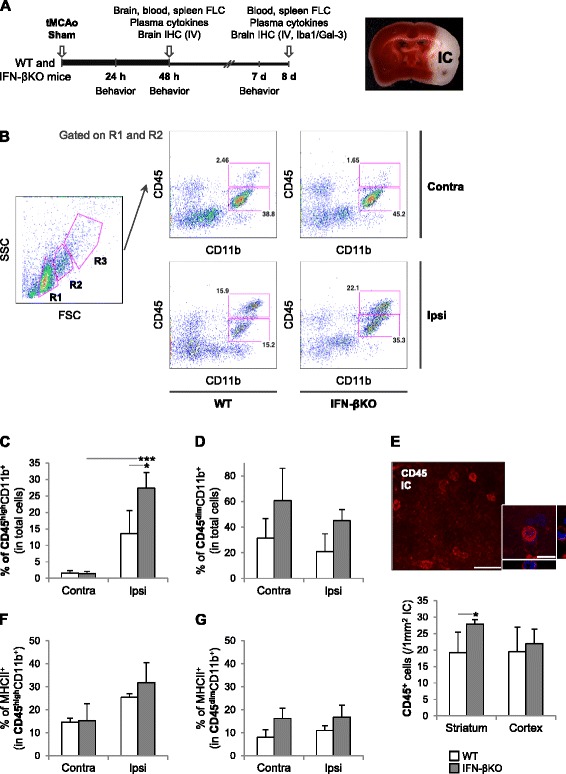
Abrogation of IFN-β in mice results in a higher accumulation of CD45^high^CD11b^+^ leukocytes within the brain 2 days after tMCAo. **a** *Left panel* experimental design: IFN-βKO mice and WT controls underwent tMCAo or sham surgery; behavioral testing was done 24 h, as well as 2 and 7 days after surgery; samples were obtained 2 and 8 days after surgery. *Right panel* photograph of a TTC-stained brain slice (WT mouse) 24 h after tMCAo reflecting the stereotypical infarct core (white). *FLC* flow cytometry, *IC* infarct core, *IHC* immunohistochemistry, *IV* infarct volume. **b**–**g** Two days post-occlusion, immune and inflammatory cells were extracted from contralateral (*Contra*) and ipsilateral (*Ipsi*) brain hemispheres (WT, *n* = 3 with three brains per *n*; IFN-βKO mice, *n* = 3 with three to four brains per *n*), and different subsets were characterized by flow cytometry analysis. **b** Representative dot plots showing gates used to discriminate CD11b^+^ cells expressing high or low levels of CD45, as well as respective changes across Contra/Ipsi hemispheres and WT/IFN-βKO animals (as indicated in the figure). Populations: *R1* (putative lymphocytes and NK cells), *R2* (putative monocytes/macrophages), and *R3* (putative granulocytes). *FSC* forward scatter, *SSC* side scatter. **c** Percentage of CD45^high^CD11b^+^ cells, (infiltrating) leukocytes, and **d** CD45^dim^CD11b^+^ cells, (brain-resident) microglia. **e** Micrographs denoting the abundance and typical morphology of CD45^+^ (Cy3, *red*) cells within the infarct core (striatum) 2 days after tMCAo (WT animal); DAPI counterstain (represented in *blue*). Scale bars, 50 μm (*left panel*) and 10 μm (*right panel*). Number of CD45^+^ cells per mm^2^ in WT and IFN-βKO mice. **f**–**g** Percentages of MHCII^+^ (activated) CD45^high^CD11b^+^ leukocytes and MHCII^+^ (activated) CD45^dim^CD11b^+^ microglia, respectively. *p < 0.05 and **p < 0.01 (Bonferroni correction)

To investigate the effect of endogenous IFN-β expression on the inflammatory response, along with the development of sensorimotor deficits and infarct volume that follow an ischemic stroke, we made use of a genetically modified mouse line, lacking the IFN-β encoding gene. These mice along with WT counterparts underwent tMCAo or sham surgery (the experimental outline is included in Fig. 1a). Putative alterations in cerebral vasculature and rCBF owing to the genetic manipulation were addressed in two ways. First, mice of both genotypes (IFN-βKO and WT) were perfused with a gelatinous carbon black solution, brains were imaged under standard conditions, and the configuration of the circle of Willis and middle cerebral artery (MCA) was analyzed. Abrogation of IFN-β in mice did not lead to detectable changes in the configuration of the circle of Willis or MCA (Additional file 1). Secondly, rCBF values at the MCA occlusion and reperfusion times were similar between genotypes, suggesting an unaltered rCBF in response to ischemia (*n* = 21–24 for each group). Occlusion values (in percentage of baseline): 18.4 ± 5.7 % (WT) and 21.2 ± 6.4 % (IFN-βKO). Reperfusion values (in percentage of baseline): 91.2 ± 16.0 % (WT) and 82.8 ± 26.0 % (IFN-βKO).

Innate immune cells (notably granulocytes, monocytes/macrophages, and NK cells) are within the first wave of immune cells recruited to the site of damage and inflammation [31]. Whether, in stroke, this process is critically regulated by endogenous IFN-β remains unclear. To address this question, we isolated immune and inflammatory cells from the brains of IFN-βKO and WT mice 2 days after tMCAo and quantified, using flow cytometry, the percentages of CD45^high^CD11b^+^ cells, (infiltrating) leukocytes, and CD45^dim^CD11b^+^ cells, (brain-resident) microglia [2, 5, 32]. Our gating strategy is demonstrated in Fig. 1b. Using this approach, we found a higher percentage of CD11b^+^ leukocytes in the ipsilateral hemispheres of IFN-βKO versus WT mice (Fig. 1c). Note that, as expected, the percentage of CD11b^+^ leukocytes is overall higher in ipsilateral than in contralateral hemispheres (*p* < 0.001, two-way ANOVA). By contrast, the percentage of CD11b^+^ microglia did not differ among examined conditions (Fig. 1d). These results indicate that, in mice lacking IFN-β, the stroke-induced accumulation of blood-originating cells is enhanced. Indeed, analysis of CD45^+^ cell numbers, in situ, revealed an approximately 50 % increase within the proximal (i.e., striatal) infarct core in IFN-βKO mice (Fig. 1e). CD11b^+^ leukocytes and microglia were analyzed further for the expression of major histocompatibility complex II (MHCII), a hallmark of APCs activation [16]. We found no significant difference in the percentage of MHCII^+^ cells within the CD45^high/dim^CD11b^+^ populations between IFN-βKO and WT mice (Fig. 1f, g), likely reflecting an equivalent leukocyte/microglial activation capacity (irrespective of the total numbers of CD45^high/dim^CD11b^+^ cells). On the other hand, a higher total number of CD45^high^CD11b^+^ leukocytes (as shown in Fig. 1c, d) and a similar percentage of MHCII^+^ cells within this cell population collectively imply a higher total number of activated leukocytes in IFN-βKO versus WT brains.

Innate immune cells include two different NK1.1-expressing subsets: NK cells (CD3^−^NK1.1^+^) and NKT (CD3^+^NK1.1^+^) cells [33]. Increases in innate cells subpopulations could result in a differential recruitment or local regulation of T cells. However, we did not find genotypic differences in NK, NKT, or T cell subsets studied—CD3^+^CD4^+^ T_h_, CD4^+^CD25^+^ T regulatory (T_reg_), CD3^+^CD8^+^ T cytotoxic (T_C_), and CD8^+^CD122^+^ T_reg_ cells (data not shown).

### The development of the Gal-3 response over the first week post-stroke is similar in IFN-βKO mice and WT controls

To explore further the possible influence of IFN-β on the development of the macrophages and microglial response post-stroke, we analyzed cerebral Iba-1 and Gal-3 immunoreactivities in IFN-βKO and WT mice, 8 days after tMCAo. While Iba-1 is generally found in macrophages and microglia, there is evidence, at least up to 72 h after stroke, that Gal-3 is preferentially expressed by activated microglia [34, 35]. The Gal-3 macrophages/microglial population is particularly interesting because it has been suggested to play a beneficial role in stroke [3, 35–37]. Gal-3^+^ cells accumulate in the infarct core over the first week after tMCAo and are responsive to the expression of inflammatory clues [4].

**Fig. 2:**
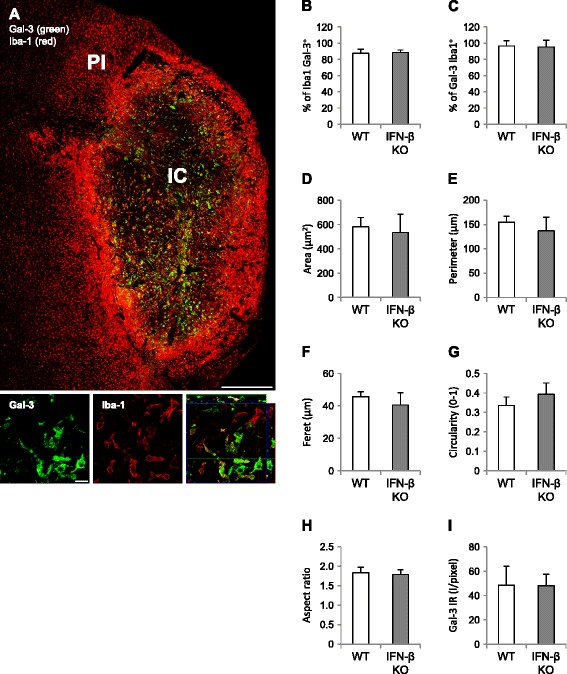
Lack of IFN-β expression does not affect cerebral Gal-3 immunoreactivity within the first 8 days after tMCAo. **a** *Top panel* representative composite micrograph of Gal-3 (Alexa488, *green*) and Iba-1 (Cy3, *red*) immunoreactivities within the injured cerebral hemisphere 8 days after tMCAo (WT mouse). *IC* infarct core, *PI* peri-infarct region. *Bottom panel* corresponding micrograph denoting Gal-3 and Iba-1 colocalization within the infarct core; note that colocalization is partial. Scale bars, 500 and 20 μm, respectively. **b** Percentage of Iba-1 colocalizing with Gal-3 (87.4 ± 0.05 % for WT mice) and **c** Gal-3 colocalizing with Iba-1 (96.5 ± 0.07 % for WT mice). **c**–**h** Area (μm^2^), perimeter (μm), circularity (0–1), Feret’s diameter (μm), and aspect ratio of Gal-3^+^ cells within infarct core, respectively. **i** Gal-3 immunoreactivity (I/pixel). **a**–**i** WT, *n* = 6–7; IFN-βKO: *n* = 7–8

Iba-1 immunoreactivity was particularly high at the outer border of the infarct core and peri-infarct region (Fig. 2a), and expectedly, Iba-1^+^ cells appeared ramified in regions remote to the infarct core and amoeboid-like close to or in the infarct core. Gal-3, on the other hand, was found predominantly within the infarct core, and Gal-3^+^ cells exhibited an amoeboid morphology. Within the infarct core of WT mice, about 87.4 ± 0.05 % of Iba-1 colocalized with Gal-3 (Fig. 2b) and 96.5 ± 0.07 % of Gal-3 colocalized with Iba-1 (Fig. 2c), findings that were similar in IFN-βKO mice. Note that most Gal-3^+^ cells express Iba-1, whereas Iba-1 itself clearly relates to a broader population of cells. Area, perimeter, Feret’s diameter as well as circularity of Gal-3^+^ cells did not differ significantly between IFN-βKO versus WT mice, suggesting a similar morphology (Fig. 2d–h). Gal-3 immunoreactivity did not differ between groups (Fig. 2i), indicative of similar expression levels/total number of cells.

Collectively, our data suggest an increased accumulation of leukocytes in the brain parenchyma of IFN-βKO mice within the first days after tMCAo, which could occur, at least in part, through a relatively increase in BBB permeability. However, we found no evidence for alterations in BBB integrity between WT and IFN-βKO mice 2 days after tMCAo, as evaluated by in situ IgG detection, reflecting vessel extravasation, or claudin-5 immunoreactivity, probing for tight junction function (Additional file 2).

### IFN-βKO mice show a higher number of circulating immature B cells after tMCAo

**Fig. 3:**
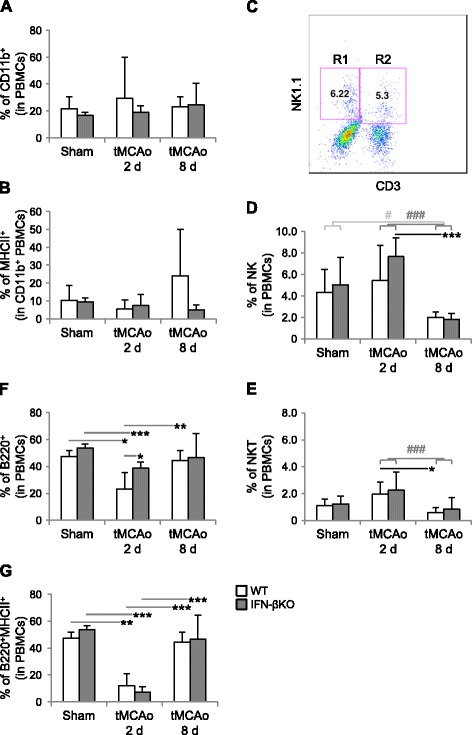
Mice lacking IFN-β show an increase in the fraction of circulating B220^+^ (immature) B cells 2 days after tMCAo. PBMCs were isolated 2 days after tMCAo (WT, *n* = 9; IFN-βKO, *n* = 11), as well as 8 days after tMCAo (WT, *n* = 6–7; IFN-βKO, *n* = 7–8) and sham surgery (*n* = 4 for each genotype). Inflammatory and immune cell subsets were analyzed by FLC. **a** Percentages of CD11b^+^ and **b** MHCII^+^ (activated) CD11b^+^ cells within PBMCs. **c** Dot plot illustrating gating for CD3^−^NK1.1^+^ versus CD3^+^NK1.1^+^ cells (NK and NKT cells, respectively). **d** Percentage of NK and **e** NKT cells within PBMCs. **f** Percentages of B220^+^ (immature) and **g** B220^+^MHCII^+^ (mature and activated) B cells in the PBMCs pool. *p < 0.05, **p < 0.01, and **p < 0.001 (Bonferroni correction). Note that stroke led to a decrease in the percentages of NK and NKT cells 8 days after tMCAo in relation to sham surgery (#*p* < 0.05, two-way ANOVA, for NK cells) and 2 days post-occlusion (##*p* < 0.001, two-way ANOVA, for NK and NKT cells)

To study the potential involvement of IFN-β in the regulation of peripheral immune cell subsets post-stroke, we isolated peripheral blood mononuclear cells (PBMCs) 2 days after tMCAo, as well as 8 days following tMCAo and sham surgery, and we quantified different immune cell subsets using flow cytometry (see Fig. 1a for the experimental outline). Whereas lack of IFN-β expression resulted in a higher percentage of CD11b^+^ leukocytes in the brain following tMCAo (Fig. 1), the percentages of different innate immune cell subsets within PBMCs were similar between genotypes, for all of the evaluated conditions (Fig. 3a–e). Irrespective of the genotype, stroke led to a decrease in the percentages of NK (gated R1) and NKT (gated R2) cells, which was observed 8 days after tMCAo, in relation to both sham surgery (*p* < 0.05, two-way ANOVA, for NK cells only) and 2 days post-occlusion (*p* < 0.001, two-way ANOVA, for NK and NKT cells), (Fig. 3c–e).

Unlike the innate immune subsets tested, the B cell response post-stroke was strikingly affected by IFN-β expression. Specifically, in WT mice, the percentage of B220^+^ B cells in the PBMC pool was lower 2 days after tMCAo than after sham surgery, returning to sham equivalent levels within 2 to 8 days after tMCAo (Fig. 3f). In IFN-βKO mice, the total percentage of B220^+^ B cells within PBMCs 2 days after tMCAo was, although reduced when compared to sham surgery, significantly higher than that in respective WT controls, and it did not differ from that observed 8 days after tMCAo. For both genotypes, stroke induced a transient decrease in B220^+^MHCII^+^ cells. However, while the percentage of B220^+^ B cells was higher in the IFN-βKO group 2 days after tMCAo, the percentage of mature and activated B220^+^MHCII^+^ cells did not differ between genotypes in any of the conditions tested (Fig. 3g). These results suggest a higher rate of proliferation of B cells in IFN-βKO mice compared to WT controls. With respect to CD3^+^CD4^+^ T_h_, CD4^+^CD25^+^ T_reg_, CD3^+^CD8^+^ T_C_, and CD8^+^CD122^+^ T_reg_cells, we did not find differences among experimental conditions (Additional file 3).

### IFN-βKO mice do not show a stroke-induced decrease in the number of splenocytes

**Fig. 4:**
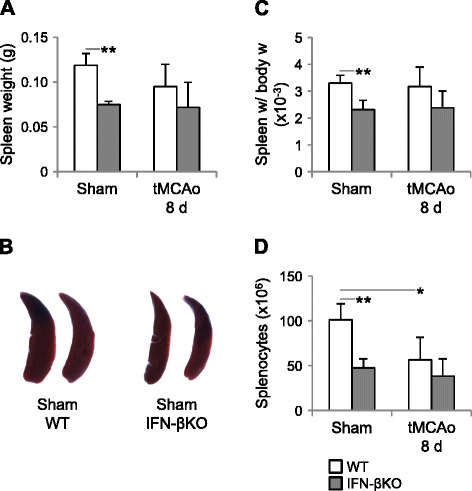
Deletion of *Ifnb* in mice decreases spleen weight and number of splenocytes under control conditions, but it prevents a stroke-induced reduction in splenocytes 8 days after tMCAo. **a** Spleen weight (g) 8 days after tMCAo (WT, *n* = 7; IFN-βKO, *n* = 8) and sham surgery (*n* = 4 for each genotype). **b** Representative photographs of spleens of sham-operated WT and IFN-βKO mice; photographs were acquired using a MicroPublisher 3.3 RTV CCD camera (QImaging, Surrey, BC, Canada), under standard conditions. **c** Spleen weight/body weight (ratio). **d** Corresponding number of splenocytes following occlusion (WT, *n* = 5; IFN-βKO, *n* = 6) and sham surgery (*n* = 4 for each genotype). *p < 0.05 and **p < 0.01 (Bonferroni correction)

Stroke-induced immunosuppression encompasses spleen atrophy, which occurs partially through apoptotic loss of splenocytes [7, 8, 10, 11] and partially through mobilization of splenic inflammatory and immune cell pools [38, 39]. For that reason, we studied the effect of IFN-β signaling on spleen weight and number of splenocytes 8 days after tMCAo or sham surgery. Surprisingly, we found that the spleens of sham-operated IFN-βKO mice weighed significantly less and were noticeably smaller than those of sham-operated WT mice (Fig. 4a, b). This phenomenon was verified further by normalizing spleen weight to body weight (Fig. 4c, see also Additional file 4). Interestingly, the tendency toward a lower spleen weight after tMCAo observed in WT animals (Fig. 4a) was not apparent after normalization (Fig. 4c). In line with the previous results, the number of splenocytes was markedly lower in sham-operated IFN-βKO versus WT mice (Fig. 4d). Moreover, we obtained a significantly lower number of splenocytes in WT mice post-occlusion versus sham surgery, and this stroke-induced decrease in the number of splenocytes did not occur in IFN-βKO mice. It is important to note that both genotypes showed similar numbers of splenocytes 8 days after tMCAo.

### Endogenous IFN-β affects stroke-induced changes in immune and inflammatory cell subsets in the spleen

**Fig. 5:**
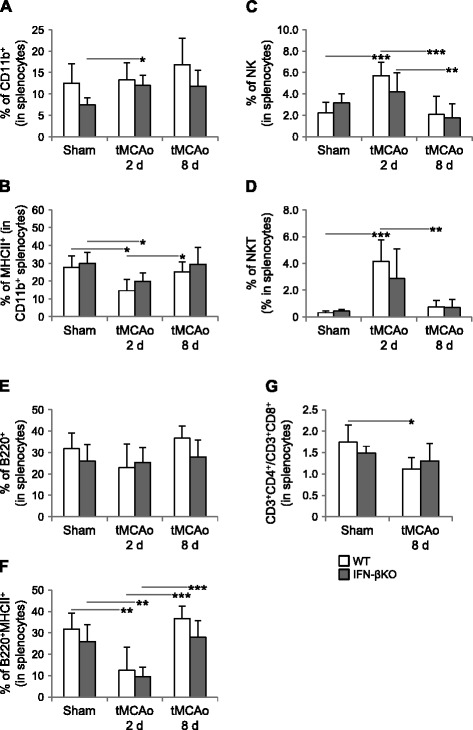
Stroke induced changes in immune and inflammatory cell subsets in the spleen are affected by the expression of IFN-β. In parallel to PBMCs, we isolated splenocytes. **a** Percentages of CD11b^+^ and **b** MHCII^+^ (activated) CD11b^+^ cells within splenocytes. **c** Percentage of NK and **d** NKT cells within splenocytes. **e** Percentages of B220^+^ (immature) and **f** B220^+^MHCII^+^ (mature and activated) B cells in the spleen. **g** Ratio of splenic CD3^+^CD4^+^ T_h_ and CD3^+^CD8^+^ T_C_ cells based on the FCT analysis (WT, *n* = 6–7, IFN-βKO, *n* = 7–8). *p < 0.05, **p < 0.01, and **p < 0.001 (Bonferroni correction)

Next, we assessed different immune and inflammatory cell subsets within splenocytes, as described previously for PBMCs. In contrast to WT controls, IFN-βKO mice showed a significant increase in the percentage of CD11b^+^ cells in the spleen 2 days after tMCAo versus sham surgery (Fig. 5a). For both genotypes, the expression of MHCII^+^ CD11b^+^ leukocytes was lower 2 days after tMCAo than that after sham surgery and, at 8 days after tMCAo, did not differ from that observed in sham-operated animals (Fig. 5b). These results suggest a differential regulation of CD11b^+^/MHCII^+^ CD11b^+^ in mice lacking IFN-β. Note also that these results contrast the MHCII^+^ CD11b^+^ profile obtained for PBMCs (Fig. 4b) and suggest a stroke-dependent modulation of MHCII regulatory mechanisms in the spleen, despite spleen atrophy. With respect to NK.1.1-expressing cell subpopulations, important stroke-dependent effects that were evident in WT mice were not reproduced in the IFN-βKO group (Fig. 5c, d). Indeed, in WT mice, the NK and NKT cell fractions increased 2 days post-occlusion versus sham, normalizing 8 days after tMCAo. In IFN-βKO mice, on the other hand, the percentage of NK cells did not increase significantly 2 days post-occlusion compared to that in sham, and the percentage of NKT cells was not significantly altered across conditions (sham, 2 and 8 days after tMCAo). These data point toward a differential regulation of NK/NKT cells in mice lacking IFN-β.

Contrasting with the increase found in PBMCs (Fig. 3), the percentage of B220^+^ lymphocytes did not differ between mice lacking IFN-β and WT controls (Fig. 5f). Yet, for both genotypes, stroke induced a transient decrease in B220^+^MHCII^+^ lymphocytes. Thus, despite spleen atrophy, a stroke-induced increase in proliferating B cells also could be verified. Finally, we evaluated the ratio between CD3^+^CD4^+^ and CD3^+^CD8^+^ T cells—lower ratios typically indicate a weakened immune system and are associated with more frequent infections [9]. This ratio was lower 8 days after tMCAo when compared to sham surgery for WT controls, but it did not change significantly in IFN-βKO mice (Fig. 5g). Thus, stroke-induced reductions in splenocyte numbers and CD3^+^CD4^+^/CD3^+^CD8^+^ T cell ratio were absent in IFN-βKO mice. The percentages of CD3^+^CD4^+^ T_h_, CD4^+^CD25^+^ T_reg_, CD3^+^CD8^+^ T_C_, and CD8^+^CD122^+^ T_reg_ cells did not differ among experimental conditions (Additional file 3).

### IFN-β KO mice show reduced IL-1β and IL-5 plasma concentrations after tMCAo

**Fig. 6:**
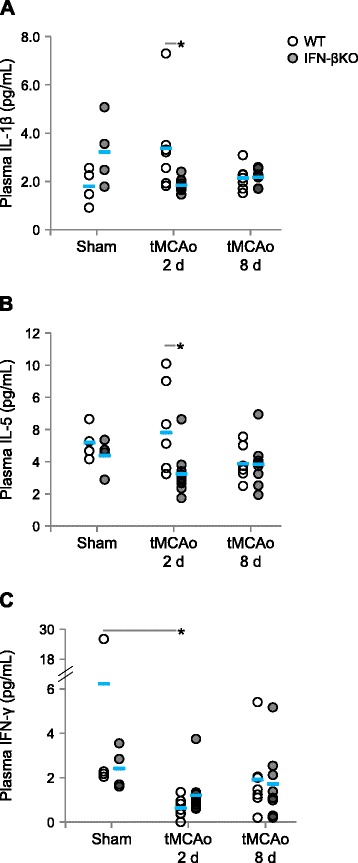
IFN-βKO mice show lower IL-1β and IL-5 plasma concentrations 2 days after tMCAo. **a**–**c** IL-1β, IL-5, and IFN-γ protein concentrations (pg/mL) in the blood plasma 2 and 8 days after tMCAo (WT, *n* = 7; IFN-βKO, *n* = 8–10), respectively; sham-operated animals served as controls (*n* = 4 for each genotype). We present individual data points and the mean (*blue line*). *p < 0.05 (Bonferroni correction)

The observed changes in immune cell populations could be accompanied by changes in plasma cytokine levels. Moreover, under inflammatory conditions, IFN-β may promote a T_h_1-T_h_2 shift [13]. Therefore, we quantified the protein concentrations of T_h_1 and T_h_2 cytokines in the plasma of IFN-βKO mice and WT controls 2 and 8 days after tMCAo/sham surgery. We found lower IL-1β (Fig. 6a) and IL-5 (Fig. 6b) levels in mice lacking IFN-β 2 days after tMCAo. Finally, while in WT mice, IFN-γ levels decreased 2 days after tMCAo versus respective sham surgery; in IFN-βKO mice, IFN-γ levels were unchanged (Fig. 6c). The genotype did not influence IL-2, IL-4, IL-10, IL-12, mKC, and TNF-α levels (Additional file 5).

### Lack of IFN-β signaling resulted in a reduction in body weight and decrease in grip strength after stroke

**Fig. 7:**
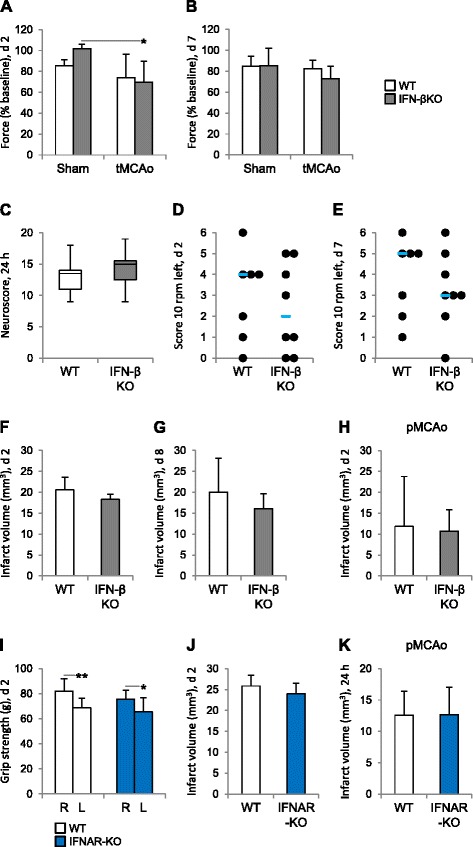
Mice lacking IFN-β show a reduction in grip strength 2 days after tMCAo. **a**–**h** IFN-βKO mice. **i**–**k** IFNAR-KO mice. **a**–**b** Grip strength (in percentage of baseline) 2 and 7 days after tMCAo (WT, *n* = 6–7; IFN-βKO, *n* = 8) and sham surgery (*n* = 4 for each genotype). **c** Composite neuroscore (0–32) 24 h after tMCAo (WT, *n* = 19; IFN-βKO, *n* = 16). **d**–**e** Rotating pole scores, referent to 10 rpm to the left, 2 and 7 days after tMCAo. Here, we present individual data points and the median (*blue line*). **f** Infarct volume (in mm^3^) 2 days after tMCAo (*n* = 5 for each genotype) and **g** 8 days after tMCAo (WT, *n* = 7; IFN-βKO, *n* = 8). **h** Infarct volume (in mm^3^) 2 days after pMCAo (*n* = 5 for each genotype). **i** Grip strength (g) for the right (*R*) and left (*L*) paws 2 days after tMCAo (WT, *n* = 7; IFNAR-KO, *n* = 8). **j** Infarct volumes at 2 days after tMCAo (WT, *n* = 6; IFNAR-KO, *n* = 8) and **k** 24 h after pMCAo (WT, *n* = 10; IFNAR-KO, *n* = 9). *p < 0.05 and **p < 0.01 (Bonferroni correction)

Along with its effect on the inflammatory response, abrogation of IFN-β could lead to important differences in physiological parameters, including weight and body temperature post-stroke. Body weight, in particular, is an important outcome measure in neurological conditions. To no surprise, stroke significantly affected both weight and body temperature (*p* < 0.01 and *p* < 0.001, respectively, mixed model ANOVA), and two main differences were apparent between IFN-βKO and WT mice. First, while no significant weight loss was noted in the WT group, IFN-βKO mice showed a higher weight loss 2 and 3 days after tMCAo than after sham surgery, (Table 1). In WT mice, body temperature decreased significantly 24 h after stroke (tMCAo compared to sham surgery); this decrease was not verified in IFN-β KO mice (Table 1). In addition to addressing physiological parameters, we evaluated sensorimotor performance through multiple behavioral tests, including the grip strength test, by which we observed a decrease in forepaw grip strength 2 days post-occlusion in the IFN-βKO group only (Fig. 7a, b). We did not find any significant difference between the two genotypes when using the composite neuroscore (24 h after tMCAo, Fig. 7c) or the rotating pole test (2 and 8 days after tMCAo, Fig.7d, e). Nonetheless, in the rotating pole test, spontaneous recovery was evident for both groups. When determining infarct volumes 2 and 8 days after tMCAo, we did not find a significant effect of IFN-β abrogation (Fig. 7f, g). Survival rates within 2 and 8 days after tMCAo did not differ between groups (in total, 7 of 29 WT and 1 of 26 IFN-βKO mice died within 2 days; 3 of 10 WT and 1 of 9 IFN-βKO mice died within 8 days); with respect to sham-operated animals, mortality was zero. To exclude a potential model bias, IFN-βKO and WT mice also were subjected to pMCAo and allowed to recover for 2 days. Consistently, infarct volumes of IFN-βKO mice were similar to that of WT controls (Fig. 7h).

**Table 1:** Physiological parameters of WT and IFN-βKO mice

Parameter	Time point	Sham	Sham	tMCAo	tMCAo
		WT	IFN-βKO	WT	IFN-βKO
Temperature (°C)	24 h	36.9 ± 0.4	36.5 ± 0.6	36.1 ± 0.4*	36.3 ± 0.6
	2 days	37.6 ± 0.1	38.0 ± 1.2	36.7 ± 0.9	36.3 ± 1.2
	3 days	37.9 ± 0.5	38.1 ± 1.2	37.4 ± 0.3	37.2 ± 0.6
	7 days	38.2 ± 0.2	38.3 ± 0.3	37.4 ± 1.0	37.5 ± 1.2
Weight loss (% baseline)	24 h	8.9 ± 0.8	8.1 ± 4.2	10.0 ± 2.2	10.2 ± 2.1
	2 days	9.4 ± 2.0	6.3 ± 4.7	13.6 ± 5.2	14.7 ± 3.0**
	3 days^a^	6.8 ± 2.6	5.4 ± 4.2	15.0 ± 5.5	16.9 ± 3.3*
	7 days	8.9 ± 2.8	5.0 ± 5.0	11.2 ± 8.6	16.2 ± 11.7

^a^Weight loss ceases between days 3 and 4 after tMCAo

*p < 0.05 (versus respective sham)

**p < 0.01 (versus respective sham)

IFN-β acts through the IFNAR. Thus, to investigate further the impact of endogenous IFN-β signaling in stroke outcome, we subjected IFNAR-KO mice and WT counterparts to tMCAo and evaluated behavior and infarct volumes 2 days after. Physiological parameters, including body temperature and weight loss were similar between IFNAR-KO and WT mice, and we did not find a significant genotype-related difference in behavioral performance after tMCAo, as assessed by the grip strength test. Nevertheless, tMCAo caused a decrease in grip strength when using the contralateral paw, demonstrating a stroke-induced asymmetry in grip strength (Fig. 7i). In line with our findings in IFN-β KO mice, the infarct volumes of IFNAR-KO mice were similar to that of WT controls both 48 h after tMCAo (Fig. 7j) and 24 h after pMCAo (Fig. 7k). Moreover, mortality did not differ significantly between groups (4 of 11 WT and 1 of 10 IFNAR-KO mice died).

## Discussion

In rodents, the first week post-stroke is thought to constitute a critical period for the formation of the infarct core and activation of mechanisms that promote or repress recovery, in both of which inflammation plays a major role [1]. Although the critical impact of inflammation on stroke outcome is well accepted, a unified view on underlying molecular and cellular determinants remains to be established. Given its reported anti-inflammatory capacity and use in the treatment of relapsing-remitting MS, IFN-β has been put forward as a candidate drug for acute stroke treatment. However, the inflammatory response post-stroke is highly dynamic, both spatially and temporally, and the precise modes of action and effects IFN-β in stroke remain largely unexplored. First, literature reporting on the therapeutic potential of IFN-β in stroke is sparse and controversial [18–22]. Secondly, the function of endogenous IFN-β signaling, which may enable a better understanding of underlying pathophysiological mechanisms and thus a potentially higher success in designing the future treatment of stroke, had not been explored.

We provide evidence that endogenous IFN-β regulates the post-stroke inflammatory response, in the brain and periphery, and we reveal its specific actions. Previous studies have consistently shown that CD11b^+^ cells found within the brain parenchyma can express relatively high or low levels of CD45, the first including infiltrating leukocytes (notably monocyte-derived macrophages) and the second resident microglia [2, 5, 32]. In C57BL/6 mice, CD45^high^CD11b^+^ cells accumulate in the brain parenchyma within the first days after focal cerebral ischemia [2, 4]. Our results indicate that endogenous IFN-β signaling limits the local accumulation of blood-originating cells that occurs after stroke. Indeed, following tMCAo, IFN-βKO mice showed a higher percentage of CD45^high^CD11b^+^ cells in ipsilateral hemispheres, and a higher number of CD45^+^ cells within the infarct core than their WT counterparts. This finding is in line with previous studies showing that, in rats, administration of rIFN-β consistently reduced the number of leukocytes in the brain up to 7 days after stroke, despite the usage of different administration protocols [19, 21]. The percentage of CD45^high^CD11b^+^ cells expressing the activation marker MHCII did not differ significantly between the IFN-βKO and WT groups. Yet, given the larger percentage of CD45^high^CD11b^+^ cells, these data likely reflect a higher total number of CD45^high^CD11b^+^MHCII^+^ cells in the absence of IFN-β. In contrast to its effects on CD45^high^CD11b^+^ cells, abrogation of IFN-β did not result in concomitant changes in the percentages of CD45^dim^CD11b^+^ cells or respective MHCII expression. Albeit the debate surrounding the exact nature and dynamics of the macrophages/microglial response after stroke, the lack of an increase in the relative numbers of microglia and activated microglia 2 days after tMCAo (Fig. 2d) probably reflects a delay in the accumulation and activation of the microglial population at the injury site. Indeed, the dramatic increase in the expression of Iba-1 and Gal-3 observed at 8 days after tMCAo is unlikely to result predominantly from invading macrophages [4]. This delay might be related, at least in part, to the extent of cerebral damage [10, 12, 30, 40]. We also show that Iba-1 and Gal-3 immunoreactivities 8 days after stroke induction are unaltered by the abrogation of IFN-β, suggesting that, in stroke, IFN-β rather acts by diminishing a relatively earlier leukocytic central invasion. Indeed, it is likely that the enhanced accumulation of leukocytes observed in the brains of IFN-βKO reflects an increase in the infiltration of these cells into the brain parenchyma. This could occur via a relative decrease in BBB integrity, through an enhanced activity of metalloproteinase-9 [19]. However, when analyzing IgG and claudin-5 signals, reflecting vessel extravasation and tight junction function, respectively, in the infarct core and/or peri-infract region, we did not find evidence for alterations in post-stroke BBB integrity induced by knocking out the gene encoding IFN-β, at least not 2 days after tMCAo. Yet on the other hand, it has been previously reported that, during EAE, IFN-βKO mice show an increase in the levels of circulating chemokines (namely CCL3 and CCL5), which also could underlie an increased leukocytic infiltration into the brain parenchyma in comparison to their WT counterparts [41]. Similarly, lack of IFNAR in mice has been shown to relate to an increase in leukocyte numbers and metalloproteinase-9 expression, as well as to a decrease in the chemoattractant CXCL10 in the CNS under inflammatory conditions [42]. Nevertheless, as indicated by a recent report, local regulatory mechanisms exerted by endogenous IFN-β [43], such as controlling local proliferation and activation of innate immune cells, cannot be excluded at present.

In addition to attenuating central inflammation, our results indicate that IFN-β is important in regulating peripheral immune cell subsets. Most notably, IFN-βKO mice showed a higher total percentage of circulating B220^+^ (immature) B cells, but similar percentages of circulating B220^+^MHCII^+^ (mature and activated) B cells in relation to WT counterparts, 2 days after tMCAo. This set of data indicates that the ratio of proliferative B cells is lower in WT mice, which is consistent with the general anti-proliferative role of IFN-β. In fact, the anti-proliferative and pro-apoptotic actions of IFN-β are well documented [15, 43]. In support of a previous report [15], no major T_h_1-T_h_2 shift was detected between genotypes. Interestingly, however, IL-1β and IL-5 plasma concentrations decreased in the IFN-βKO group, and these cytokines have been extensively associated with multiple aspects of B cell function, including proliferation [44, 45]. Although we found similar numbers of splenocytes and splenic B cells in WT and IFN-βKO 2–8 days after tMCAo, higher numbers of circulating B cells also could be due, at least in part, to an increased mobilization of these cell population from splenic or other (notably thymic) pools. It is worth noting that the decrease in circulating B220^+^ B cells following tMCAo might be in agreement with previous studies showing a stroke-induced apoptotic loss of B lymphocytes [7, 11].

Besides its effects on circulating immune cells, we also provide evidence for a role of IFN-β in the regulation of splenic immune cell subsets. Indeed, the IFN-βKO group did not show a stroke-induced decrease in the total number of splenocytes, and multiple observations point toward a differential regulation of CD11b^+^, NK, NKT, and T cells in the presence and absence of endogenous IFN-β. First, only IFN-βKO mice showed a significant increase in the percentage of CD11b^+^ cells in the spleen 2 days after tMCAo (compared to sham surgery). In line with B cell results, this data possibly reflects a higher proliferative capacity of CD11b^+^ cells in IFN-βKO mice. Second, in the IFN-βKO group, stroke did not lead to a transient increase in NK cells (otherwise observed in WT mice) or to significant changes in the percentage of NKT cells. Third, a stroke-induced reduction in the CD3^+^CD4^+^/CD3^+^CD8^+^ T cell ratio was absent in IFN-βKO mice. Abrogation of IFN-β also led to a lack of suppression of IFN-γ levels 2 days after tMCAo. The described peripheral changes converge toward an overall deficient immunoregulation in the IFN-βKO group, which could explain the increased brain inflammation. It is worth noting that while sham-operated IFN-βKO mice did have smaller spleens, and that deletion of IFN-β was previously shown to cause structural alterations, along with a decrease in resident macrophages in the spleen [46], we did not find significant shifts in immune cell populations in the spleen (or blood). Moreover, at least a certain degree of responsiveness of IFN-β splenocytes (particularly CD11b^+^ cells) to the post-stroke milieu appears to have been preserved.

In relapsing-remitting EAE, endogenous IFN-β signaling is sufficient to limit inflammation and promote a better disease outcome [15]. Here, IFN-β KO mice, in contrast to their WT counterparts, showed a significant weight loss, one of the most accepted consequences of neuroinflammatory conditions (such as EAE), and a significant reduction in grip strength 2–3 days after stroke. Using other behavioral approaches, different time points after stroke induction, and the receptor knockout, we could not detect further differences in sensorimotor deficits. Also, we show, not only at the level of the cytokine but also at the level of the receptor and using two models of stroke, tMCAo and pMCAo, that endogenous IFN-β signaling does not influence infarct volume 2 to 8 days after stroke. On the one hand, and albeit having found no difference in body temperature between IFN-βKO mice and WT controls, a potential differential effect of our post-surgical care strategy on infarct volume cannot be ruled out at present. In addition, differences in the extent of sensorimotor deficits or in the functional recovery curve may have been masked by the sensitivity of behavioral tests. Yet on the other hand, these results are consistent with two previous studies showing no effect of IFN-β administration within the first 3–7 days following ischemic stroke [21], and more recently, subarachnoid hemorrhage [22]. We hypothesize that this uncoupling between inflammatory changes and, to some extent, outcome is due to the multiple actions of IFN-β. While it may limit the accumulation of immune and inflammatory cells in the brain, it may also, via anti-proliferative and pro-aptoptotic actions contribute to stroke-induced peripheral immunosuppression. In fact, it also is becoming increasingly clear that, within the injured CNS, inflammatory and immune cells may play both pro- and anti-inflammatory roles [47]. A CNS injury-induced peripheral immunosuppression may serve to diminish a potentially harmful immune response toward the brain, particularly given the exposure of self-antigens [48], but a certain degree of autoimmunity may be beneficial [49]. Moreover, peripheral immunosuppression has been associated to an increased susceptibility to infections, which constitutes a common cause of death after cerebral ischemia [9, 12].

## Conclusions

We conclude that endogenous IFN-β signaling critically regulates inflammation, and it may positively impact acute stroke outcome. Our study evidences the complexity of inflammatory alterations provided by lack of a specific inflammatory mediator following stroke, which should be taken into account in the development of immunomodulatory therapies.

## Additional files

Additional file 1:
**Middle cerebral artery configuration.** Configuration of the middle cerebral artery in WT (right) and IFN-βKO (left) mice, upon perfusion with a gelatinous carbon black solution. Representative photographs. (PDF 100 kb) Additional file 2:
**Similar IgG and claudin‐5 signals in WT and IFN‐βKO brains 2 days after tMCAo.** **a** Image of the IgG-related signal observed in coronal brain slices, 2 days after tMCAo, showing extravasation of IgG in and around the infarct area, in WT and IFN-βKO mice, as indicated in the figure. *IC* infarct core. Scale bar, 200 μm. **b** Representative image of claudin-5^+^ vessels in the peri-infarct area. **c** The bar graph shows the percentage of intact vessels covering the peri-infarct region (1 mm^2^) in each of the genotype groups (*n* = 5, *p* = 0.46, Student’s *t* test). **d** Graph denoting the average length of vessels in the peri-infarct region in WT and IFN-βKO mice (*n* = 5, *p* = 0.25, Student’s *t* test). (PDF 89 kb) Additional file 3:
**Characterization of different populations of T lymphocytes in the blood and spleens of WT and IFN‐βKO mice during the first week post‐surgery.** **a** CD3^+^CD4^+^ (Th cells). **b** CD4^+^CD25^+^ (Treg cells). **c** CD4^+^CD8^+^ (TC cells). **d** CD8^+^CD122^+^ (Treg). **a-b** *Left panel* PBMCs. *Right panel* splenocytes. We present the results as percentage of total cells analyzed and as mean ± SD. (PDF 86 kb) Additional file 4:
**Complete profile of WT and IFN‐βKO mice spleen weight across conditions.** Spleen weight normalized to body weight in sham-operated mice (*n* = 4 for each genotype), 2 days after tMCAo (WT, *n* = 9; IFN-βKO, *n* = 11) and 8 days after tMCAo (WT, *n* = 7; IFN-βKO, *n* = 8). **p* < 0.05 and ***p* < 0.01 (Bonferroni correction). (PDF 57 kb) Additional file 5:
**Expression of Th1 and Th2 cytokines in the blood plasma of WT and IFN‐βKO mice during the first week post‐surgery.** **a-d** Plasma IFN-γ, IL-2, IL-4, IL-10, IL-12, mKC, and TNF-α protein concentrations (pg/mL), respectively. We present individual data points and the mean (*blue dash*). (PDF 93 kb)

## Abbreviations

ANOVA: analysis of variance
APCs: antigen-presenting cells
BBB: blood-brain barrier
CD: complement of differentiation
CBF: cerebral blood flow
DAPI: 4′,6-diamidino-2-phenylindole
FLC: flow cytometry
Gal-3: galectin-3
Iba-1: ionized calcium-binding adapter molecule 1
IFN: interferon
IFN-βKO: interferon-β knockout
IFNAR: IFN-α/β receptor
IFNAR-KO: IFN-α/β receptor knockout
IL: interleukin
MHCII: major histocompatibility complex II
mK or GRO/CXCL-1: mouse keratinocyte-derived factor
NeuN: neuronal nuclei
NK: natural killer
PBMCs: peripheral blood mononuclear cells
pMCAo: permanent middle cerebral artery occlusion
T_C_: T cytotoxic
T_h_: T helper
tMCAo: transient middle cerebral artery occlusion
TNF: tumor necrosis factor
T_reg_: T regulatory
TTC: triphenyltetrazolium chloride
WT: wild type
