# Supplementary material for: Endogenous IFN-β signaling exerts anti-inflammatory actions in experimentally induced focal cerebral ischemia
Source: J Neuroinflammation. 2015 Nov 18;12:211. doi: 10.1186/s12974-015-0427-0 (PMC4652356; doi:10.1186/s12974-015-0427-0)
Supplement: Additional file 1: — Middle cerebral artery configuration. Configuration of the middle cerebral artery in WT (right) and IFN-βKO (left) mice, upon perfusion with a gelatinous carbon black solution. Representative photographs. (PDF 100 kb) [file 12974_2015_427_MOESM1_ESM.pdf]

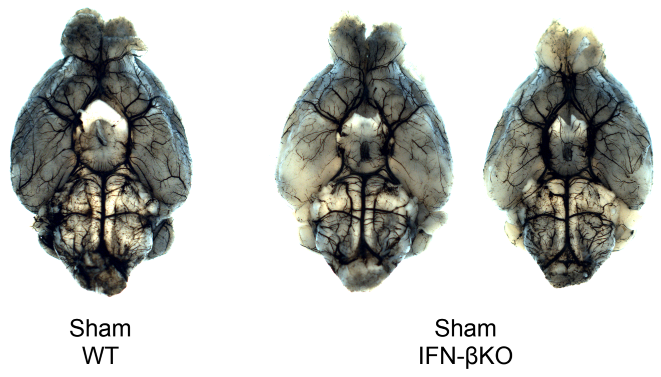

Additional file 1_Middle cerebral artery configuration. Configuration of the middle cerebral artery in WT (right) and IFN-βKO (left) mice, upon perfusion with a gelatinous carbon black solution. Representative photographs.
