# Supplementary material for: Endogenous IFN-β signaling exerts anti-inflammatory actions in experimentally induced focal cerebral ischemia
Source: J Neuroinflammation. 2015 Nov 18;12:211. doi: 10.1186/s12974-015-0427-0 (PMC4652356; doi:10.1186/s12974-015-0427-0)
Supplement: Additional file 2: — Similar IgG and claudin‐5 signals in WT and IFN‐βKO brains 2 days after tMCAo. a Image of the IgG-related signal observed in coronal brain slices, 2 days after tMCAo, showing extravasation of IgG in and around the infarct area, in WT and IFN-βKO mice, as indicated in the figure. IC infarct core. Scale bar, 200 μm. b Representative image of claudin-5+ vessels in the peri-infarct area. c The bar graph shows the percentage of intact vessels covering the peri-infarct region (1 mm2) in each of the genotype groups (n = 5, p = 0.46, Student’s t test). d Graph denoting the average length of vessels in the peri-infarct region in WT and IFN-βKO mice (n = 5, p = 0.25, Student’s t test). (PDF 89 kb) [file 12974_2015_427_MOESM2_ESM.pdf]

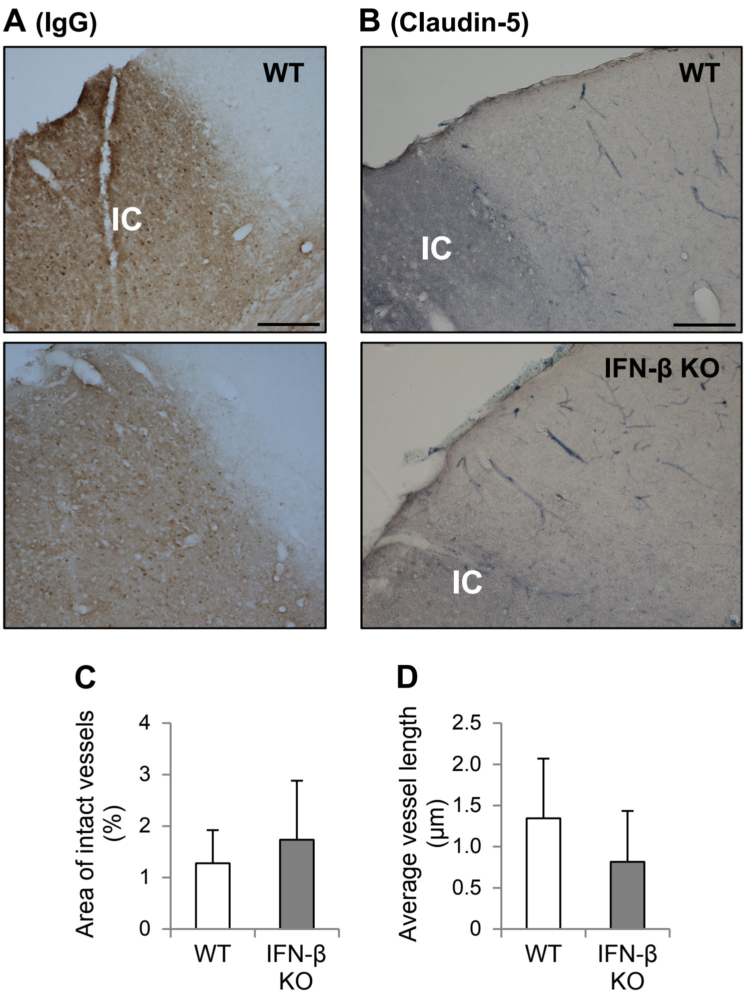

Additional file 2_Similar IgG and claudin-5 signals in WT and IFN-βKO brains 2 days after tMCAo. a Image of the IgG-related signal observed in coronal brain slices, 2 days after tMCAo, showing extravasation of IgG in and around the infarct area, in WT and IFN-βKO mice, as indicated in the figure. *IC* infarct core. Scale bar, 200 µm. **b** Representative image of claudin-5$^+$ vessels in the peri-infarct area. **c** The bar graph shows the percentage of intact vessels covering the peri-infarct region (1 mm^2) in each of the genotype groups (*n=5*, *p=0.46*, Student's *t* test). **d** Graph denoting the average length of vessels in the peri-infarct region in WT and IFN-βKO mice (*n=5*, *p=0.25*, Student's *t* test).
