# Supplementary material for: Endogenous IFN-β signaling exerts anti-inflammatory actions in experimentally induced focal cerebral ischemia
Source: J Neuroinflammation. 2015 Nov 18;12:211. doi: 10.1186/s12974-015-0427-0 (PMC4652356; doi:10.1186/s12974-015-0427-0)
Supplement: Additional file 3: — Characterization of different populations of T lymphocytes in the blood and spleens of WT and IFN‐βKO mice during the first week post‐surgery. a CD3+CD4+ (Th cells). b CD4+CD25+ (Treg cells). c CD4+CD8+ (TC cells). d CD8+CD122+ (Treg). a-b Left panel PBMCs. Right panel splenocytes. We present the results as percentage of total cells analyzed and as mean ± SD. (PDF 86 kb) [file 12974_2015_427_MOESM3_ESM.pdf]

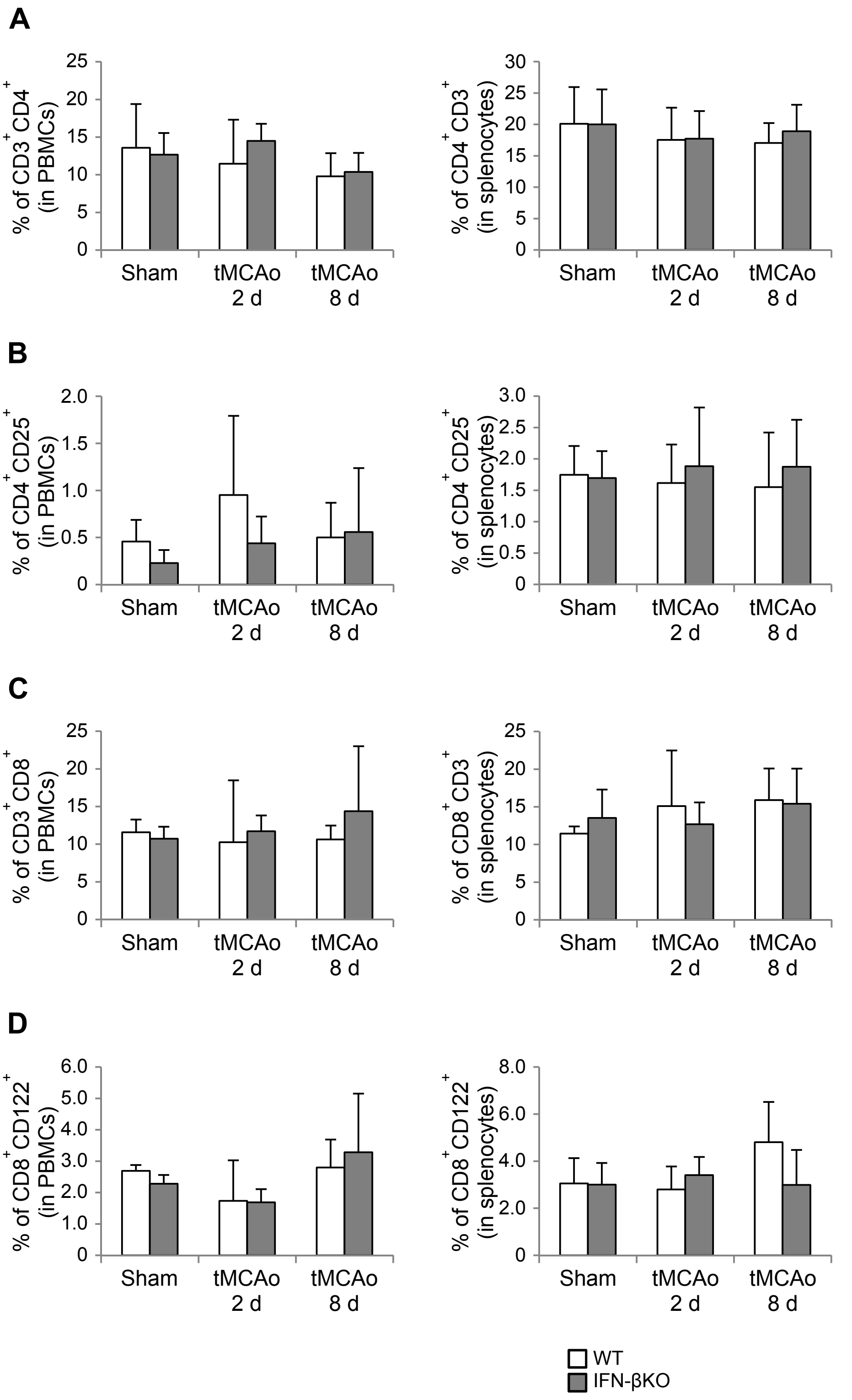

Additional file 3_Characterization of different populations of T lymphocytes in the blood and spleens of WT and IFN-βKO mice during the first week post-surgery. a $CD3^{+}CD4^{+}$ (T_h cells). **b** $CD4^{+}CD25^{+}$ (T_{reg} cells). **c** $CD4^{+}CD8^{+}$ (T_C cells). **d** $CD8^{+}CD122^{+}$ (T_{reg}). **a-b** *Left panel* PBMCs. *Right panel* splenocytes. We present the results as percentage of total cells analyzed and as mean ± SD.
