# Supplementary material for: Endogenous IFN-β signaling exerts anti-inflammatory actions in experimentally induced focal cerebral ischemia
Source: J Neuroinflammation. 2015 Nov 18;12:211. doi: 10.1186/s12974-015-0427-0 (PMC4652356; doi:10.1186/s12974-015-0427-0)
Supplement: Additional file 4: — Complete profile of WT and IFN‐βKO mice spleen weight across conditions. Spleen weight normalized to body weight in sham-operated mice (n = 4 for each genotype), 2 days after tMCAo (WT, n = 9; IFN-βKO, n = 11) and 8 days after tMCAo (WT, n = 7; IFN-βKO, n = 8). *p < 0.05 and **p < 0.01 (Bonferroni correction). (PDF 57 kb) [file 12974_2015_427_MOESM4_ESM.pdf]

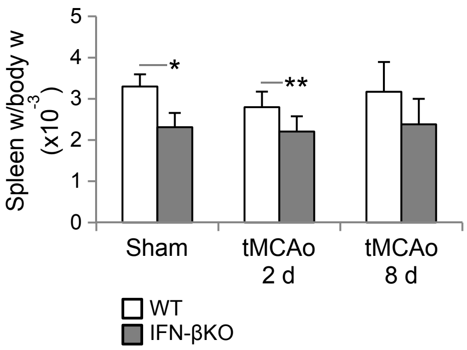

Additional file 4_Complete profile of WT and IFN-βKO mice spleen weight across conditions. Spleen weight normalized to body weight in sham-operated mice (*n=4* for each genotype), 2 days after tMCAo (WT, *n=9*; IFN-βKO, *n=11*) and 8 days after tMCAo (WT, *n=7*; IFN-βKO, *n=8*). **p<0.05* and ***p<0.01* (Bonferroni correction)
