# Supplementary material for: Endogenous IFN-β signaling exerts anti-inflammatory actions in experimentally induced focal cerebral ischemia
Source: J Neuroinflammation. 2015 Nov 18;12:211. doi: 10.1186/s12974-015-0427-0 (PMC4652356; doi:10.1186/s12974-015-0427-0)
Supplement: Additional file 5: — Expression of Th1 and Th2 cytokines in the blood plasma of WT and IFN‐βKO mice during the first week post‐surgery. a-d Plasma IFN-γ, IL-2, IL-4, IL-10, IL-12, mKC, and TNF-α protein concentrations (pg/mL), respectively. We present individual data points and the mean (blue dash). (PDF 93 kb) [file 12974_2015_427_MOESM5_ESM.pdf]

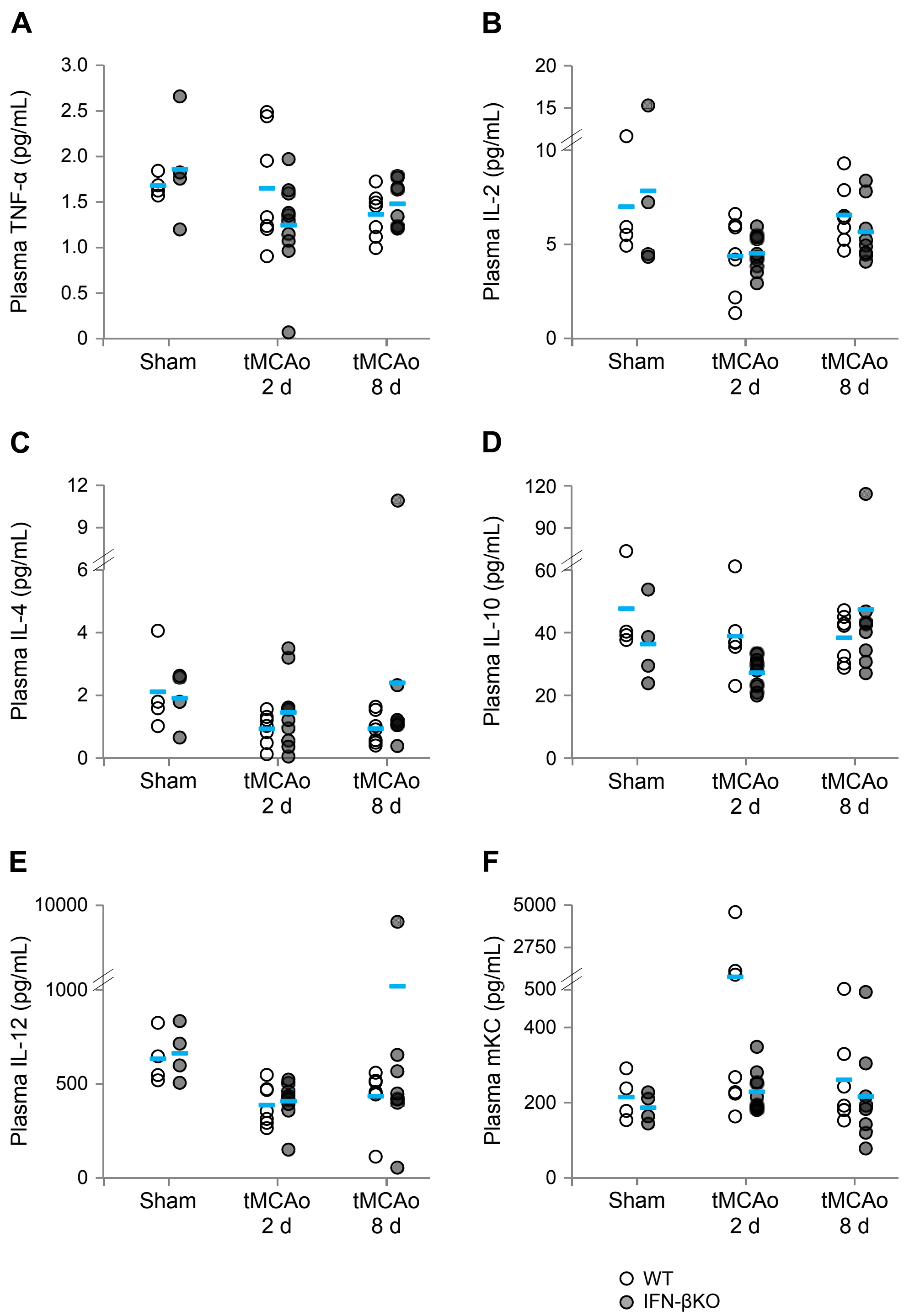

Additional file 5_Expression of Th1 and Th2 cytokines in the blood plasma of WT and IFN-βKO mice during the first week post-surgery. a-d Plasma IFN-γ, IL-2, IL-4, IL-10, IL-12, mKC and TNF-α protein concentrations (pg/mL), respectively. We present individual data points and the mean (*blue dash*).
